# Microwaves reduce water refractive index

**DOI:** 10.1038/s41598-022-15853-9

**Published:** 2022-07-07

**Authors:** Yusuke Asakuma, Tomoisa Maeda, Takahiro Takai, Anita Hyde, Chi Phan, Shinya Ito, Shuji Taue

**Affiliations:** 1 Department of Chemical Engineering, University of Hyogo, Shosha 2167, Himeji, 671-2280 Japan; 2 Department of Chemical Engineering, Curtin University, GPO Box U1987, Perth, WA 6845 Australia; 3 School of System Engineering, Kochi University of Technology, Kami, Kochi 782-8502 Japan

**Keywords:** Chemical engineering, Photochemistry, Physical chemistry, Theoretical chemistry, Optical metrology

## Abstract

Microwaves, long used as a convenient household appliance, have been increasingly used in industrial processes such as organic synthesis and oil processing. It has been proposed that microwaves can enhance these chemical processes via a non-thermal effect. Here we report the instantaneous effect of microwaves on the permittivity and phase velocity of light in water through the in-situ measurement of changes in refractive index. Microwave irradiation was found to reduce the water refractive index (RI) sharply. The reduction increased as a function of microwave power to a far greater extent than expected from the change in temperature. The phase velocity of light in water increases up to ~ 5% (RI of 1.27) during microwave irradiation. Upon stopping irradiation, the return to the equilibrium RI was delayed by up to 30 min. Our measurement shows that microwaves have a profound non-thermal and long-lasting effect on the properties of water. Further investigation is planned to verify if the observed RI reduction is restricted to the region near the surface or deep inside water bulk. The observation suggests a relationship between microwave-induced and the enhanced aqueous reactions.

## Introduction

Recently, microwaves have been employed to enhance numerous chemical processes because of the ability to achieve targeted heating conditions in the solvent^1–5^. Microwave-specific heating effects such as local hot spots likely contribute to an improved reaction rate^1,2^. Similarly, the reaction selectivity of some reaction types is drastically improved by irradiation^3,6^. The specific improvements have led to numerous studies on the existence of a “non-thermal” effect, particularly on the microwave interaction with polar solvents^7^, where molecules continuously re-align their dipole moments with the alternating field^8^. It has also been argued that the non-thermal effect can contribute to the activation energy of the reaction, for certain chemical intermediaries, as a result of the change in molecular orientation and the rotation of polar solvents^6^. Despite several proposed mechanisms, the non-thermal effect remains controversial^5^. Recently, we have shown changes to liquid–liquid interfaces^9^ and gas–liquid interfaces^10^ during microwave irradiation, in which the surface effect was related to a microwave-induced change in the hydrogen bond (H-bond) network^9^. Since the reaction rate and interfacial tension modification are closely related to the water structure, changes to this structure during irradiation clarify the non-thermal effect.

According to current data by non-equilibrium molecular dynamics simulation^4^, both translational and rotational movements are essential for the hydration behaviour when polar compounds in aqueous solutions are modelled during microwave irradiation. In this study, we employ in-situ observation of the refractive index (RI) during microwave irradiation to gauge changes to the water structure. Since the speed of light is a constant, the refractive index depends on the phase velocity of light through a medium. Water has a relatively large RI due to strong hydrogen bonds. Therefore, if the water network is affected via heating or microwave irradiation, the phase velocity of light and refractive index will change correspondingly. Moreover, the refractive index is directly linked to permittivity and permeability, both critical parameters for microwave heating^11^.

**Figure 1:**
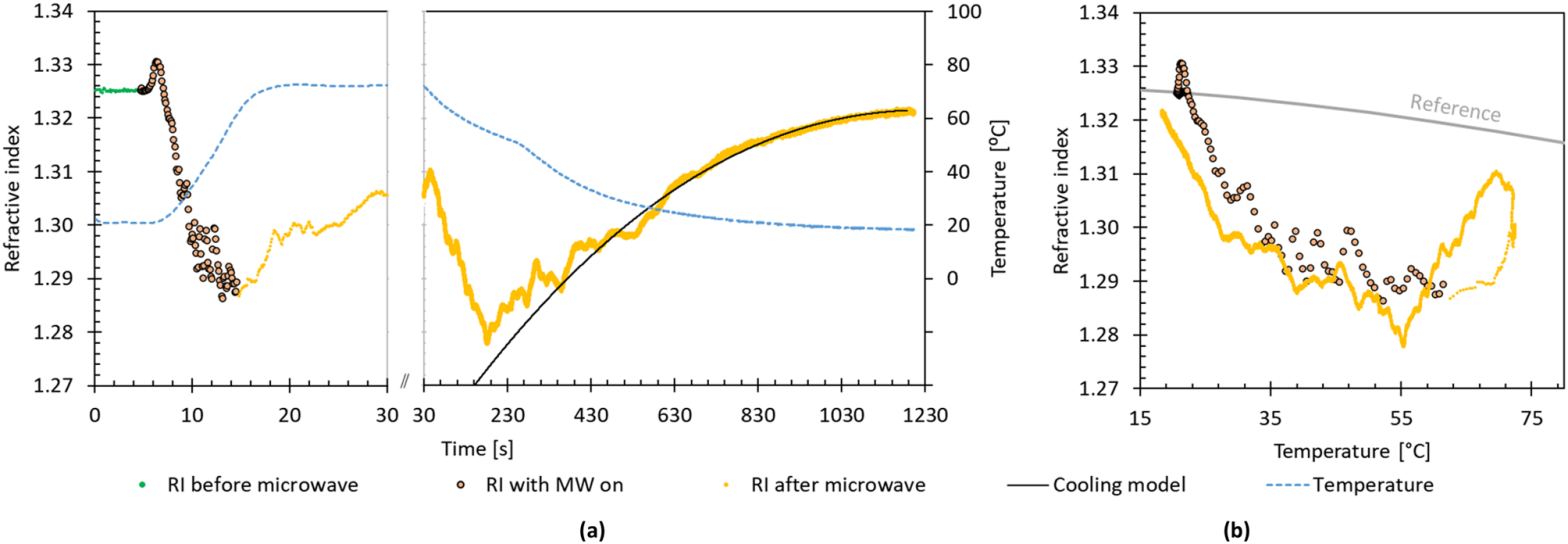
Changes to the refractive index of water when irradiated by microwaves at 400 W for 10 s. (**a**) Change in RI and temperature as the experiment progresses, showing the measured refractive index (RI), with data sampled 10 times per second. The temperature was sampled once per second and interpolated using polynomial fits. The timescale on the left is enlarged to show the rate of change during heating, and the second panel includes the cooling model for comparison. (**b**) The change in RI is shown with respect to temperature. Reference data for the RI of water is from Mitra et al.^12^*.*

Figure 1 shows the refractive index during and after the irradiation for 400 W × 10 s. The RI dropped quickly during the irradiation period. As soon as irradiation was stopped, there was a recovery in the RI, as shown in the enlarged view of Fig. 1a. There was a fluctuation period prior, including the second drop at ~ 130 s. Subsequently, the RI recovered gradually as the cell cooled. The RI reached values much lower than anticipated given the literature data for the change of water RI with temperature. For instance, the literature value for RI at 100 °C is 1.31^12^. Figure 1b shows the value of RI versus temperature during and after irradiation. In the “cooling model”, the rate of recovery in the refractive index was linked to the shrinking kinetics of nanobubbles or voids^13^, as detailed in SI. The fluctuation period right after irradiation can be attributed to thermal convection (the critical Rayleigh number corresponds to a temperature of ~ 38 °C at the surface of the thermometer probe).

**Figure 2:**
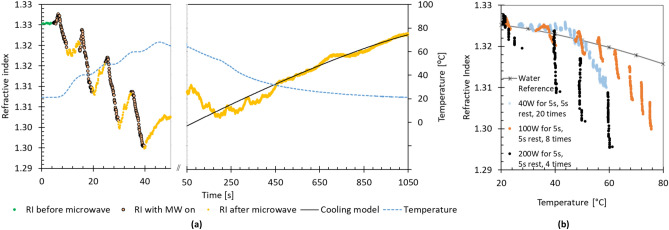
(**a**) Changes to the refractive index (RI) of water when irradiated by microwaves at 200 W in four, 5-s pulses. RI data is sampled 10 times per second, the temperature is measured at 1 s intervals and interpolated using polynomial fits. The timescale on the left panel is expanded. (**b**) Comparison of the refractive index of water measured during pulsed irradiation by 40 W, 100 W and 200 W. The pulsed patterns are adjusted to provide a total of 4000 J irradiated energy. Reference data for the RI of water is from Mitra et al.^12^.

The pulsed pattern was employed to clarify the microwave impact. The total energy was kept at 4000 J as before (200 W for 5 s, repeating four times with 5 s cooling between pulses; 8 × 5 s × 100 W pulses; 20 × 5 s × 40 W pulses). As seen in Fig. 2a,b, the effect on the RI was accumulative, with more significant effects seen with higher irradiation power. Furthermore, the initial rapid recovery in RI was observed after each pulse, after which the recovery is slow. Summarily, the in-situ data demonstrates a strong non-thermal effect of MW on water RI.

**Figure 3:**
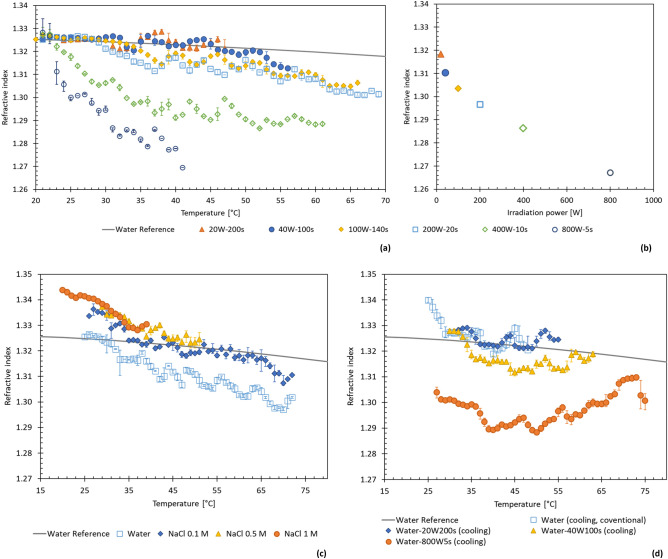
(**a**) Comparison of refractive index changing with temperature, for experiments with continuous irradiation of various microwave power. The irradiation time is changed to provide a consistent 4000 J overall. (**b**) The minimum refractive index measured for each run, showing a greater decrease in refractive index with higher irradiation power. (**c**) Refractive index measured with different salt solutions. Each solution was made up to 0.1 M and irradiated with 200 W for 20 s. For (**a,c**), markers show the average RI at *1* °C intervals, with the error bars indicating the range of the measured data. (**d**) RI data as a function of temperature during cooling after irradiation of 4000 J at 20 W, 40 W and 800 W. Reference data for the RI of water is from Mitra et al.^12^.

Depression of the RI increased as the microwave power was increased from 200 to 800 W, with the irradiation time adjusted to maintain total irradiation energy of 4000 J. Figure 3a shows the refractive index of water as it is irradiated with microwaves of different power, with more significant depression when irradiated with a higher power as per Fig. 3b. Finally, the measurement was repeated with different NaCl concentrations, as shown in Fig. 3c. The electrolyte has a weak but positive influence on RI, due to the influence of ions on the water H-bonds network^14^.

## Discussion and conclusion

The refractive index of a medium is defined as the ratio of the speed of light through a vacuum to the speed of light through the medium. The reduction in RI observed during the experiments indicates that the phase velocity of light in water during irradiation is faster than that without irradiation.

**Figure 4:**
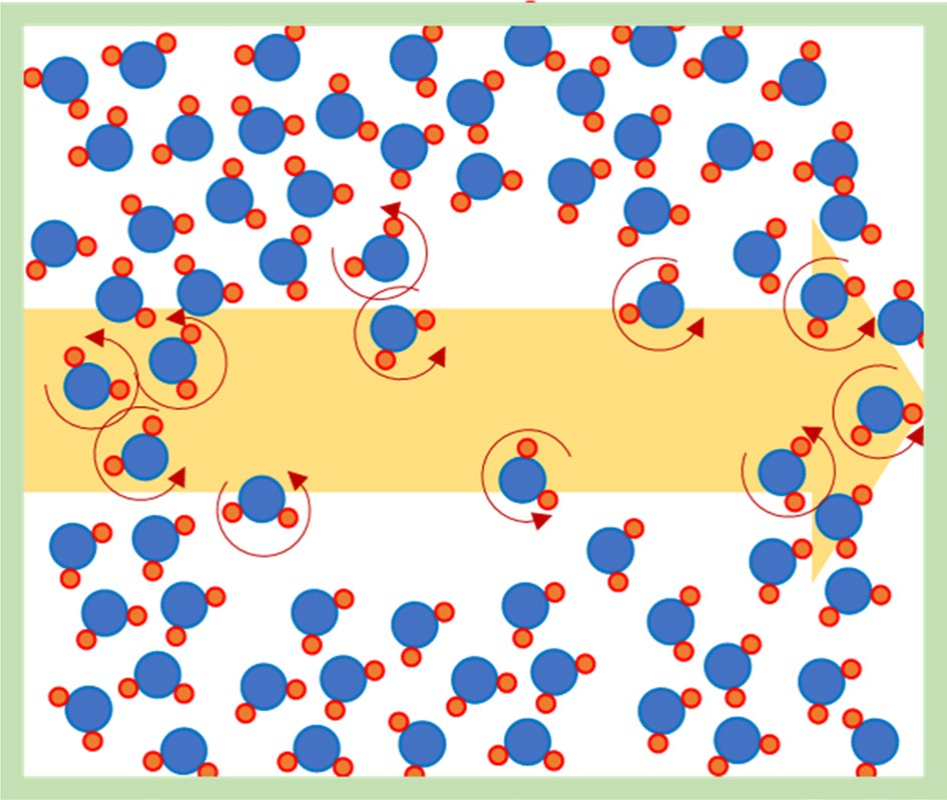
Voids formation during microwaves, caused by increasing water molecular rotation and disruption of H-bonds.

Quantitatively, the RI is a function of electric permittivity, ε, and magnetic permeability, μ. It is well-known that static water permittivity decreases with increasing temperature^15^. In addition to heating, under microwave irradiation, the water bonding network collapses due to strong molecular rotation, which can enhance reaction kinetics^16^. The molecular rotation can form voids or nanobubbles of vapour within the liquid body. This is shown conceptually in Fig. 4. The water complex permittivity was also reduced with increasing temperature in the microwave frequency range^17^. It has been shown that water has reduced permeability in confined nano-space^18^ due to a lack of space for water-water H-bonds. A previous study with GHz-THz spectroscopy has validated the monotonic reduction of water RI with increasing frequency^19^.

The data in this study shows that in addition to a thermal (heating) effect, microwaves directly reduced the RI of the solution. The relation between refractive index and temperature in Fig. 3b,d show the extent of reduction in RI varied with MW power, even when the total irradiated energy was kept constant. The depressed RI was evident during the cooling phase (Fig. 1b). Collectively, the results demonstrate a non-equilibrium phenomenon. The delayed recovery can be explained by considering H-bonds reorientation and voids/nanobubbles formation. It is well-known that the average number of water H-bonds decreases with increasing temperature^20,21^. The reorientation of the H-bonds network should be the main controlling factor during microwave irradiation, as demonstrated by the sharp and linear reduction in Figs. 1a and 2a. As seen in Fig. 3a, the RI value can be reduced to 1.27, which means the phase velocity of light increases by ~ 5%. High molecular rotation can also generate localized voids or nanobubbles (Fig. 4). Although degassed water is used in this experiment, nanobubbles or voids might be formed due to localized vaporization observed with plasmonic bubbles^13^. The voids may also attach to the quartz surface after microwaving and prolong the recovery. In our previous study, microwave-induced bubbles on nanoparticles were also confirmed by dynamic light scattering^22^. As the H-bonds relaxation is in order of picoseconds^21^, the shrinking of voids dominates the recovery curve after microwaves. We are working on the measurement to verify the surface nanobubbles/voids. Further experiments with different aqueous solutions might also verify if the observed RI reduction is restricted to the region near the surface or deep inside water bulk.

In summary, the optic measurement of water during and after microwave irradiation shows that microwave-induced RI does not follow the temperature-dependency. Instead, the observed data indicated a non-thermal effect on the water properties. The behaviour strongly depends on microwave power and accumulates with repeating irradiation. Depending on the power, the effect can last for hours after the irradiation. The dynamic effect can be modelled from the dynamic shrinking of voids. The observation can explain the enhanced aqueous reactions during microwave irradiation.

## Methods

Regarding the measurement of the refractive index, the optical fibre of SMS (Single-Multi-Single) structure consisting of both ends and the centre, as shown in Fig. S1a is used^11,23^. Because the fibre is made of quartz, with low electromagnetic resistance, it is suitable for measurement during microwave irradiation. As shown in the enlarged view of the fibre wall surface in Fig. S1b, multiple modes are reflected in the central part, and they interfere with each other. At this time, the change of the refractive index (*n*_2_) in aqueous solution near the outside of the fibre is detected as the change in the penetration depth, *z*. Finally, the refractive index is calculated from the depth. The detailed theory is described in our previous studies^11,23^.

Figure S2 shows the measurement system of refractive index during the microwave irradiation, and the equipment consists of light (1552 nm), detector (PDA50B2, Thorlabs Inc.), computer, glass cell with optical fibre and microwave device (2.45 GHz: Micro Denshi Co., Ltd.). Deionized and degassed water (8 mL) is added into the cylinder solvent container, 130 mm by 12 mm (outside diameter), through which the fibre passes. The fibre diameter is 0.125 mm. The container with the fibre, is placed at the centre of the microwave reactor (290 mm × 110 mm × 50 mm). The temperature fibre (FS100, Anritsu meter Co., Ltd.) is inserted from the top, and the tip of the temperature probe is placed near the optical fibre. The reflector at the end of the waveguide is adjusted to maximize the solvent temperature during the irradiation. Finally, temperature and refractive index are monitored during and after the irradiation. The measurement continued until the temperature reached 30 °C with natural cooling. Various types of microwave modes are irradiated. Microwave power varies at 20, 100, 200, 400 and 800 W. The irradiation time was determined by fixing the irradiation energy at 4000 J.

## Supplementary Information

Supplementary Information.
