# Supplementary material for: Microwaves reduce water refractive index
Source: Sci Rep. 2022 Jul 7;12:11562. doi: 10.1038/s41598-022-15853-9 (PMC9262909; doi:10.1038/s41598-022-15853-9)
Supplement: Supplementary file 1 — Supplementary Information. [file 41598_2022_15853_MOESM1_ESM.docx]

Microwaves reduce water refractive index

Yusuke ASAKUMA ^a^, Tomoisa MAEDA ^a^, Takahiro TAKAI ^a^, Anita HYDE ^b^, Chi PHAN ^b^, Shinya ITO ^c^ and Shuji TAUE ^c^

^a^ Department of Chemical Engineering, University of Hyogo, Shosha 2167, Himeji, 671-2280, Japan

^b^ Department of Chemical Engineering, Curtin University, Perth, WA 6845, Australia

^c^ School of System Engineering, Kochi University of Technology, Kami, Kochi 782-8502, Japan

Email: [asakuma@eng.u-hyogo.ac.jp](mailto:asakuma@eng.u-hyogo.ac.jp)

**Methods**

| 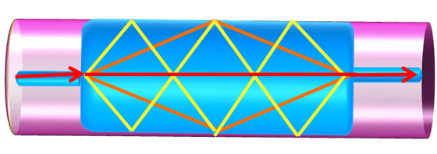 | Solvent  Fibre  *z*  *θ*  *n*_1_  *n*_2_ |
| --- | --- |
| 1. Optical fibre of SMS structure | (b) Interface between solvent and fibre |
| **Fig. S1.** Relation between penetration depth and refractive index of solvent outside fibre | |

| Light 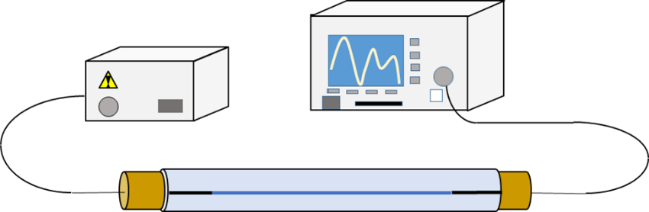 Cylinder solvent container  Optical fibre  Detector and PC | 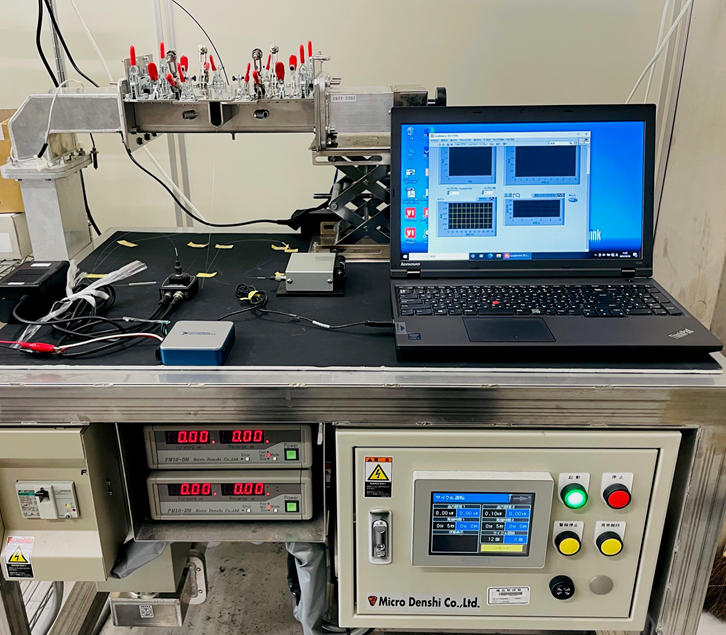  Reactor |
| --- | --- |
| (a) Schematic diagram of the experimental system | Overview of system with microwave devise and controller |

| 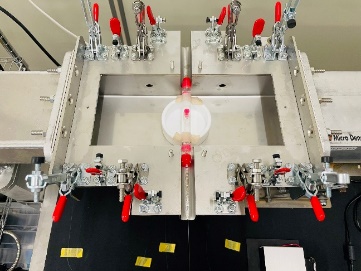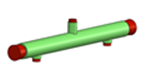 *φ*12  130  container  Size of waveguide reactor  (W:290 D:110 H:50) | 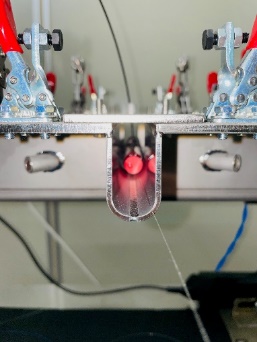 Fibre  (temperature)  Fibre  (refractive index)  container |
| --- | --- |
| (c) Size and structure of the solvent container | (d) Enlarged view near the container with two fibres in the reactor |
| **Fig. S2.** Measurement system of refractive index during microwave irradiation (dimensions are in mm). | |

**Modelling the RI recovery**

The modelling of the RI after the microwave is based on the formation of the voids or nanobubbles. These voids are formed when water molecules vaporise locally. The shrinkage dynamic of voids is similar to that of plasmonic bubbles :

$R\left( t \right)\propto\left( t_{0}-t \right)^{\alpha}$ (1)

Where *R*(*t*) is the transient radius of the voids and α is the scaling factor. Assuming the measured RI is the sum of water and voids, according to the volumetric faction:

$RI(t)={{RI}_{v} C}_{v}\frac{3}{4}\pi\left( t_{0}-t \right)^{3\alpha}+{RI}_{\infty}$ (2)

Where *RI_v_* and *RI_∞_* are refractive index of void and water at the room temperature; and *C_v_* the initial void concentration.

The above equation can be simplified as:

$RI(t)=C_{1}\left( t_{0}-t \right)^{3\alpha}+{RI}_{\infty}$ (3)

Where *C_1_* at is a constant which accounts for the gas RI and the voids initial concentration.
